# Supplementary material for: State-of-the-Art Review: Technical and Imaging Considerations in Hybrid Transcatheter and Minimally Invasive Left Ventricular Reconstruction for Ischemic Heart Failure
Source: J Clin Med. 2022 Aug 18;11(16):4831. doi: 10.3390/jcm11164831 (PMC9409787; doi:10.3390/jcm11164831)
Supplement: Supplementary file 1 [file jcm-11-04831-s001.zip › jcm-1799654-supplementary.pdf]

Supplementary data

Supplementary Table S1. Echocardiographic data of hybrid left ventricular reconstruction

**Reference**	**Naar 2021**	**Klein 2019**	**Loforte 2019**	**Klein 2019**	**Wang 2021**
Patients (n)	23	35	7	9	26
**LVEF (%)**	Baseline: 32 ± 7<br><br>6 months: increase* (p=0.13, n=20)<br><br>2 years: increase* (p=0.01, n=18)<br><br>5 years: increase * (p=0.46, n=11)	Baseline: 30 ± 8<br><br>12 months: 36 ± 6 (p=0.412, n=11)	Baseline: 22.8 ± 8.1<br><br>Discharge: 35 ± 7.2 (p=0.001, n=7)	Baseline: 28 ± 8<br><br>Directly postoperatively: 40 ± 10 (+43%, p<0.001)	Baseline: 36 ± 9<br><br>9 months: 46 ± 10 (p<0.001)
**LVESVI (ml/m²)**	Baseline: 73 ± 27<br><br>6 months: 51.5 ± 22 (-30%, p<0.001)<br><br>2 years: 49.9 ± 20 (-33%, p<0.001)<br><br>5 years: 56.1 ± 16 (-31%, p=0.047)	Baseline: 75 ± 32<br><br>12 months: 50 ± 12 (p<0.001, n=11)	Baseline: 93.2 ± 10.5<br><br>Discharge: 52.1 ± 15.1 (p<0.001, n=7)	Baseline: 53 ± 8<br><br>Directly postoperatively: 30 ± 11 (-43%, p<0.001)	Baseline: 85 ± 26<br><br>9 months: 66 ± 24
**LVEDVI (ml/m²)**	Baseline: 107 ± 27<br><br>6 months: decrease* (p<0.001, n=20)<br><br>2 years: decrease* (p<0.001, n=18)<br><br>5 years: decrease* (p=0.04, n=11)	Baseline: 110 ± 39<br><br>12 months: 78 ± 19 (p=0.015, n=11)	Baseline: 137.2 ± 20.1<br><br>Discharge: 78 ± 10.2 (p=0.001, n=7)	Baseline: 75 ± 23<br><br>Directly postoperatively: 45 ± 6 (-40%, p=0.001)	Baseline: 108 ± 33<br><br>9 months: 91 ± 32

**Tricuspid regurgitation**	Baseline: grade 0.64 ± 0.6 (scale 0-4)<br><br>6 months: grade 1.68 ± 0.8 (p<0.001)<br><br>2 years: grade 1.18 ± 0.8 (p=0.08)<br><br>5 years: grade 1.65 ± 1.0 (p=0.003)	-	No change in 6 (86%) patients; decrease from moderate to mild in 1 (14%) patient.	Baseline: grade 0.5 ± 0.6 (scale 0-4)<br><br>Directly postoperatively: 0.7 ± 1.0<br><br>Increase in TR in 2 (22%) patients	-

Values are mean ± SD or n (%). Abbreviations: LVEF, left ventricular ejection fraction; LVEDVI, left ventricular end-diastolic volume index; LVESVI, left ventricular end-systolic volume index; -, not reported; * not specified.

Supplementary Table S2. Functional data of hybrid left ventricular reconstruction

**Reference**	**Naar 2021**	**Klein 2019**	**Loforte 2019**	**Klein 2019**	**Wang 2021**
**NYHA class**	Baseline: 2.3 ± 0.5 (n=23)<br><br>6 months: decrease * (p=0.27; n=20)<br><br>2 years: decreas e* (p=0.11; n=18)<br><br>5 years: 1.6 ± 0.7 (p=0.01; n=11)	Baseline: 2.6 ± 0.5 (n=35)<br><br>6 months: 1.8 ± 0.7 (p=0.001; n=26)<br><br>12 months: 1.7 ± 0.6 (p=0.001; n=19)	Baseline: 3.4 ± 0.6 (n=7)<br><br>190 ± 105 days: 1.4 ± 0.9 (p=0.001; n=7)	Baseline: 2.7 ± 0.4 (n=9)<br><br>Discharge: 2.3 ± 0.7 (p=0.58; n=8)	Baseline: 2.7 ± 0.6<br><br>9 months: 1.7 ± 0.7 (p<0.001; n=26)
**6-MWT**	Baseline: 381 ± 103 (n=23)<br><br>6 months: 392 ± 97 (p=ns, n=20)<br><br>2 years: 432 ± 77 (p=0.06; n=17)<br><br>5 years: - (p=ns; n=10)	Baseline: 365 ± 90 (n=33)<br><br>6 months: 426 ± 99 (p=0.004; n=23)<br><br>12 months: 450 ± 75 (p=0.002; n=17)	-	-	Baseline: 369 ± 40 m (n=26)<br><br>9 months: 462 ± 61 m (p<0.001; n=26)
**MLHFQ**	Baseline: 22 (n=23)	Baseline: 42 ± 21<br><br>6 months: 18 ± 14 (p<0.001; n=26)	-	-	-

	6 months: decrease* (p=0.82; n=19)<br>2 years: decrease * (p=0.61; n=16)<br>5 years: decrease * (p=0.91; n=10)	12 months: 26 ± 21 (p=0.001; n=18)

Values are mean ± SD. Abbreviations: MLHFQ, Minnesota Living with Heart Failure Questionnaire; NYHA, New York Heart Association; 6-MWT, six minute walk test; ns, not significant; -, not reported; *not specified.
